# The follow-up observation on the therapeutic effect of photodynamic therapy for the juxtapapillary retinal capillary hemangioma: a case report

**DOI:** 10.1186/s12886-018-0923-y

**Published:** 2018-10-26

**Authors:** Bainan Tong, Jun Xiao, Guanfang Su

**Affiliations:** Department of Ophthalmology, The Second Hospital of Jilin University, Changchun, China

**Keywords:** Juxtapapillary retinal capillary hemangioma, Photodynamic therapy, Subfoveal fluid

## Abstract

**Background:**

The present study reported a case of juxtapapillary retinal capillary hemangioma (JRCH) that was successfully treated by two sessions of full-fluence photodynamic treatment (PDT) with good visual outcome.

**Case presentation:**

A 19-year-old male patient presented progressive deterioration of the vision of right eye due to the presence of exudative macular detachment associated with JRCH. The best-corrected visual acuity (BCVA) had decreased from 1.0 to 0.02. The JRCH was treated with two sessions of full-fluence PDT at an interval of 3 months. After the first PDT, the subfoveal fluid was reduced, albeit not completely disappeared. After the second PDT, the subfoveal fluid was successfully displaced. At the 1.5-year follow-up examination, no subfoveal fluid was observed at the macula, and VA improved from a pretherapy level of 0.02–0.8 at 18 months post-treatment.

**Conclusion:**

Resolution of the exudative macular detachment, reduction in papillomacular area fluid, and reduction in the size of the JRCH were observed during the follow-up period. No severe adverse events were observed. Therefore, PDT is potential candidate treatment for relieving exudative macular detachment and recovering VA and reduction in the size of the JRCH.

## Background

A juxtapapillary retinal capillary hemangioma (JRCH) is a vascular tumor located at the border of the optic nerve head. It can occur as an isolated vascular abnormality or as a manifestation of von Hippel-Lindau (VHL) disease. JRCHs are treated if they are progressive or if the lesions affect visual acuity (VA). Several treatments have been proposed, such as radiotherapy, cryotherapy, transpupillary thermotherapy, and laser photocoagulation; however, no modality in the treatment of JRCH has been effective [1]. The photocoagulation of the tumor has been widely accepted as an optimal treatment for JRCH. However, the clinical outcome of photocoagulation has not been satisfactory because of the close proximity of the tumor to the optic disc.

Herein, we report a case of JRCH associated with exudative macular detachment that underwent successful therapy with photodynamic treatment (PDT). PDT enables a selective vascular occlusion and might be less damaging to the optic disc [2]. Some studies reported that JRCH was treated with PDT; however, the treatment effect was not distinct and presented some complications [3, 4].

## Case presentation

**Fig. 1:**
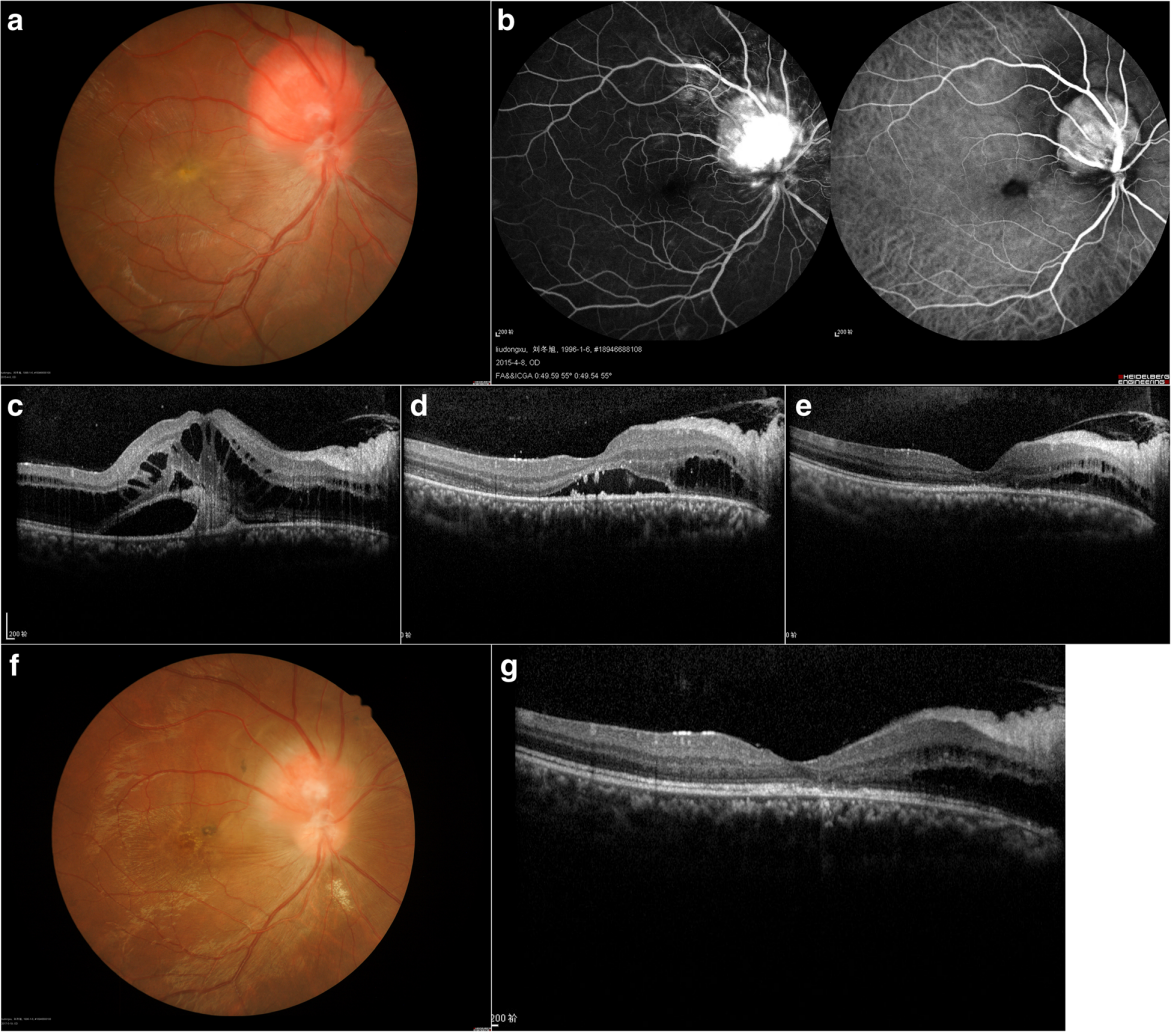
A 19-year-old male presented with a reduced right-eye vision for 6 months. **a** A reddish elevated lesion measuring 3.3 mm × 3.2 mm was revealed at the optic disc, with the surrounding SRF and exudation extended to the macular. **b** FA and ICGA demonstrated hyperfluorescence of the tumor vessels in the early phase and a continuous leakage in the late phase of the angiogram. **c** OCT revealed an extensive serous retinal detachment, and the central foveal thickness was increased to 830 μm. **d** One month after the first PDT, SRF was decrease significantly, and central foveal thickness was decreased to 277 μm. **e** One month after the second PDT, OCT revealed that the subfoveal fluid was completely abolished and the central foveal thickness was 149 μm. **f** A follow-up at 18 months and fundus examination of the right eye showed that the size of the JRCH was 2.0 mm × 1.5 mm. **g** The follow-up at 18 months: the results of OCT showed that the structure of the macular ellipsoid zone of the photoreceptors in the right eye was better than before; however, cystoid changes in the inner and outer nuclear layers were persistent

A 19-year-old male was a student and presented with a reduced right-eye vision for 6 months. The male was Han Chinese nationality. He did not have any systemic disease cand family history. The result of head magnetic resonance imaging (MRI) was normal. Systemic investigations did not show any evidence of von Hippel-Lindau disease. His best-corrected visual acuity (BCVA) was 0.02 and 1.0 for the right and left eyes, respectively. The fundus examination of the right eye revealed an elevated reddish lesion measuring 3.3 mm × 3.2 mm at the optic disc, with the surrounding subretinal fluid (SRF) and exudation extended to the macular region (Fig. 1a). No other abnormalities were found in the retina of the other eye. Fluorescence angiography (FA) and Indocyanine green angiography (ICGA) demonstrated hyperfluorescence of the tumor vessels in the early phase and a continuous leakage in the late phase of the angiogram, thereby confirming the diagnosis of JRCH (Fig. 1b). Optic coherence tomography (OCT) revealed an extensive serous retinal detachment, and the central foveal thickness was increased to 830 μm (Fig. 1c). Thus, JRCH was diagnosed.

After discussing the treatment options with the patient, standard PDT was performed using Visudyne infusion at a dose of 6 mg/m^2^ body surface area for 10 min. Subsequently, after 5 min, light exposure was performed with a diode laser (5000 μm) at 600 mW/cm^2^ for 83 s. The spot size was adjusted to encompass the largest diameter of the hemangioma without extension into the surrounding retina.

One month after the first PDT, the BCVA of the patient was 0.1 and 1.0 for the right and left eyes, respectively. The fundus examination of the right eye showed a reduced size of the JRCH, approximately measuring 3.0 mm × 2.0 mm. In addition, a significant decrease in the SRF was observed. OCT revealed that the central foveal thickness was decreased to 277 μm (Fig. 1d). After 3 months of the first PDT, the subfoveal fluid was reduced but not disappeared. Subsequently, we applied a second PDT with a spot size of 2700 μm based on the same strategy as the first session.

One month after the second PDT, the BCVA in his right eye improved to 0.2. OCT revealed that the subfoveal fluid was disappeared and the central foveal thickness was 149 μm (Fig. 1e). The structure of the macular ellipsoid zone of the photoreceptors in the right eye was discontinued, and cystoid changes in the inner and outer nuclear layers were observed. Up to the final follow-up at 18 months, the BCVA of the patient’s right eye had stabilized to 0.8. The fundus examination of the right eye showed that the size of the JRCH was 2.0 mm × 1.5 mm (Fig. 1f). The results of OCT showed that the structure of the macular ellipsoid zone of the photoreceptors in the right eye was better than before; however, cystoid changes in the inner and outer nuclear layers persisted (Fig. 1g). The final visual field examination showed an enlarged blind spot in the right eye. Presently, the patient is under continuous follow-up.

## Discussion and conclusions

To date, any single modality in the treatment of JRCH has not been effective, which might be due to the lesion located in the optic nerve. The treatment depends on the location and size of the JRCH and varies from observation to radiotherapy, cryotherapy, transpupillary thermotherapy, laser photocoagulation, PDT, antivascular endothelial growth factor (anti-VEGF) agents, vitreoretinal surgery, or combination of treatment modalities [5, 6]. If the JRCH is not associated with SRF, exudation, and vision-threatening, careful observation is recommended. Laser photocoagulation is effectively used to treat small RCH (up to 1.5 mm) in the posterior retina, but carries additional risk for JRCH due to the proximity to the optic nerve [5, 7]. Radiotherapy, cryotherapy, and transpupillary thermotherapy are commonly used to treat large JRCHs, located in the peripheral retina and away from the optic nerve. The above treatments exert a specific effect on the treatment of RCH. However, due to the proximity to the optic nerve, the treatment of JRCH usually requires multiple and intense burns and damages to the nerve fiber layer, causing a permanent scotoma and irreversible decline of the VA. Vitreoretinal surgery can also serve as an alternative when glial proliferation leads to epiretinal membrane development or tractional retinal detachment [8].

Reportedly, PDT is an alternative method to treat JRCHs as it enables a selective vascular occlusion and appears to be less damaging to the optic disc [2]. PDT might cause fibrosis and involution of the small JRCHs. In the case of large tumors, verteporfin may only be activated on the surface of the tumor, and the reactive oxygen species may not allow closure of the deeper tumor vessels [9]. Although PDT has been reported to be effective in treating macular edema and SRF in JRCH [2], it has some complications, such as retinal vessel occlusion, optic neuropathy, tractional retinal detachment, epiretinal membrane, and massive subretinal hemorrhage [10]. Bata et al. reported a case of the JRCH associated with detachment of the macular that showed massive subretinal hemorrhage and increased SRF after PDT [3]. Mariotti et al. reported a case of a progressive paramacular JRCH, with a sessile exophytic growth and associated with a tractional macular detachment that was managed successfully with a 25-gauge vitreoretinal surgery, followed by two sessions of half-fluence PDT [4].

Present, the intravitreal injection of VEGF exhibited different effects in the treatment of JRCH. The anti-VEGF therapy reduces blood vessel leakage through the change in vasoactive factors; however, there is no evidence that PDT coupled with the anti-VEGF therapy is better than single PDT, and hence, the specific effects the therapy necessitate further studies [11].

In the current case, we did not completely cover the optic nerve in order to reduce the damage to the optic nerve. The final visual field examination showed that the enlarged blind spot in the right eye was considered corresponding to the location of the tumor. The second PDT treatment was performed as a result of residual subfoveal fluid at 3 months after the first PDT. The patient was treated with single PDT. After 2 years of follow-up, the VA was recovered, hemangioma significantly reduced, SRF absorbed, and exudation and macular edema regressed, thereby proving that the photodynamic therapy in controlling the development of JRCH and in reducing the tumor and tumor exudation activity is effective.

## Abbreviations

anti-VEGF: Anti-vascular endothelial growth factor
BCVA: Best-corrected visual acuity
FA: Fluorescence angiography
ICGA: Indocyanine green angiography
JRCH: Juxtapapillary retinal capillary hemangioma
MRI: Magnetic resonance imaging
OCT: Optic coherence tomography
PDT: Photodynamic treatment
SRF: Subretinal fluid
VA: Visual acuity
VHL: Von Hippel-Lindau
